# Novel Approach to the Treatment in Rheumatic Diseases: From Molecule to Value for Patient

**DOI:** 10.1155/2018/3495782

**Published:** 2018-08-09

**Authors:** Ewa Mojs, Marek Brzosko, Wlodzimierz Samborski

**Affiliations:** ^1^Department of Clinical Psychology, Poznan University of Medical Sciences, Poznan, Poland; ^2^Department of Rheumatology, Internal Medicine and Geriatrics, Pomeranian Medical University in Szczecin, Szczecin, Poland; ^3^Department of Rheumatology and Rehabilitation, Poznan University of Medical Sciences, Poland

The treatment of rheumatic diseases changed with the appearance of biological drugs modifying the disease. This happened 20 years ago. The presence of the first anti-TNF agents, including infliximab (chimeric), adalimumab (humanized), and etanercept (soluble receptor), changes the treatment in the efficacy as well as in the aims of treatment. First, biologic treatment gave many options in choice and decisions regarding the drug and, second, the aim of treatment was the quality of life and cooperation with patients as a partner in the process. Then prognosis in these diseases is not so poor as some years ago.

Parallel to the appearance of those therapies, rheumatologists improve the management of traditional drugs, such as methotrexate, and added other nonpharmacological methods of work and therapy of patients, which gave the optimal approach toward patient and its disease.

This special issue presents the newest data in area of genetics and role of DNA and miRNA dysregulations in pathogenesis of rheumatic diseases.

The pathogenesis of lupus is contributed by genetic factors and its epigenetic modifications. miRNAs play important function in the posttranscriptional regulation of most gene-regulatory pathways and regulate both the innate and the adaptive immune responses. Dysregulation of miRNAs paths in lupus appearance is presented in the special issue. The authors show the newest data in that area.

Investigators present also work in area of genetics and answer the question whether osteopontin (OPN) variants are associated with susceptibility to ankylosing spondylitis (AS) in a Chinese population. There are interesting data polymorphisms at the 9175th position in exon 7 of OPN.

Another part of the special issue is a paper focused on orally administered small molecules, kinase inhibitors, which target blocking the pathogenic signalling process and open new way in the management of RA. A major milestone presented in the review study was the use of kinase inhibitors, tofacitinib. Author discusses the rationale for the use of kinase inhibitors in RA and shows new way of treatment with use of JAK family.

The special issue presents an article that presented socioeconomic factors which may reduce access to the biologic treatment in Romania. The data are actual not only in Romania but also in some other countries in Europe, for example, in Poland.

An interesting point in the special issue is that there are data on mud therapy as additional way of treatment in knee osteoarthritis—data presented from therapy centre in Italy.

We hope that readers will be satisfied with the modern, multidisciplinary data presented in the special issue.

*Ewa Mojs* *Marek Brzosko* *Wlodzimierz Samborski*

